# Supplementary material for: Photoreceptor calyceal processes accompany the developing outer segment, adopting a stable length despite a dynamic core
Source: J Cell Sci. 2024 Apr 12;137(7):jcs261721. doi: 10.1242/jcs.261721 (PMC11058337; doi:10.1242/jcs.261721)
Supplement: Supplementary information [file joces-137-261721-s1.pdf]

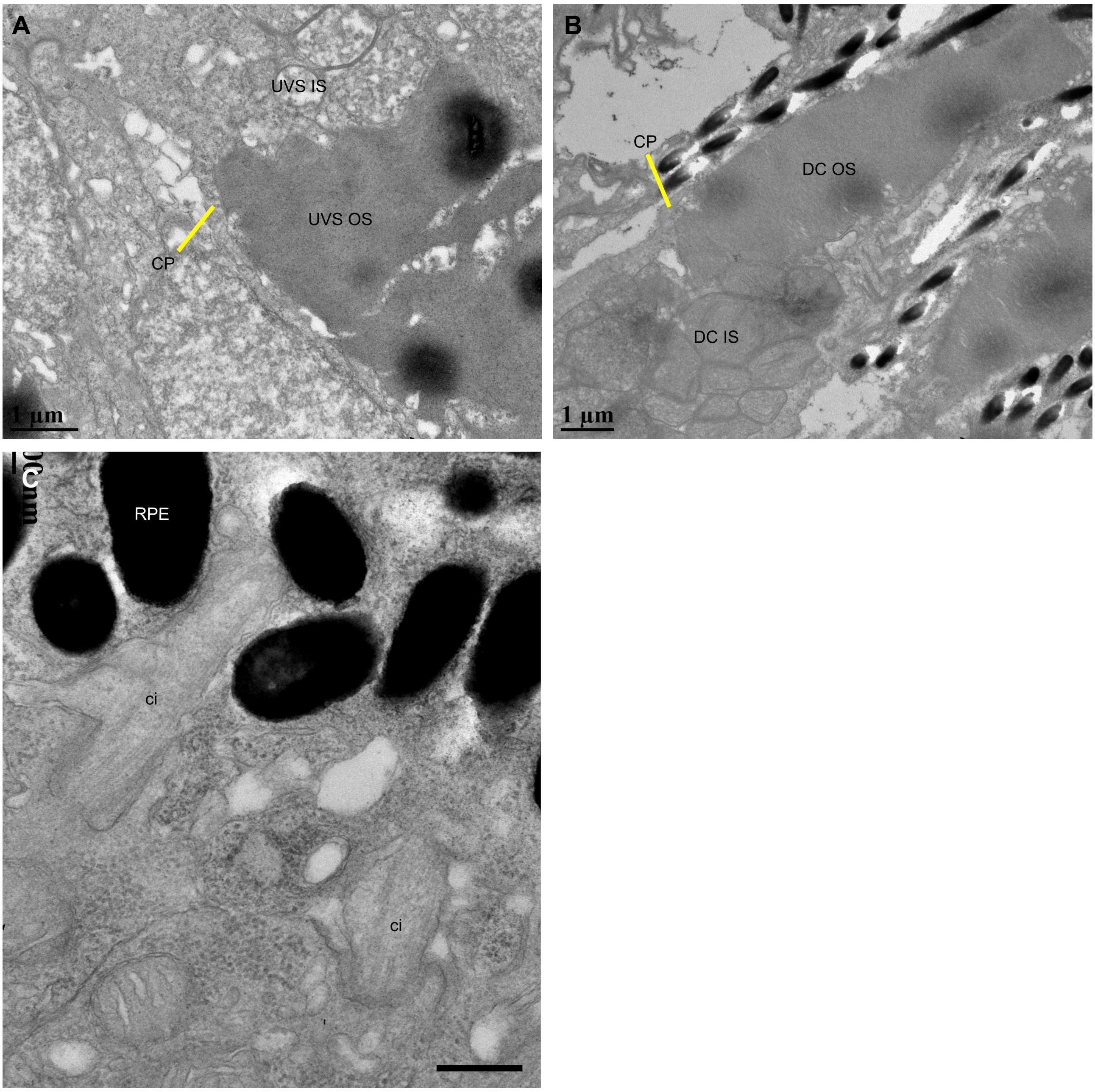

Fig. S1. (A,B) TEM micrographs of UVS cone (A) and DC (B) with CP, IS, and OS highlighted. (C) TEM micrograph of a 70 hpf central retina, enlarged area from Fig. 3C for a closer view at nascent cilia (ci). Number of fish analyzed n=5 (A,B), n=3 (C). Scale bars: 1 µm (A,B), 0.4 µm (C).

Journal of Cell Science • Supplementary information

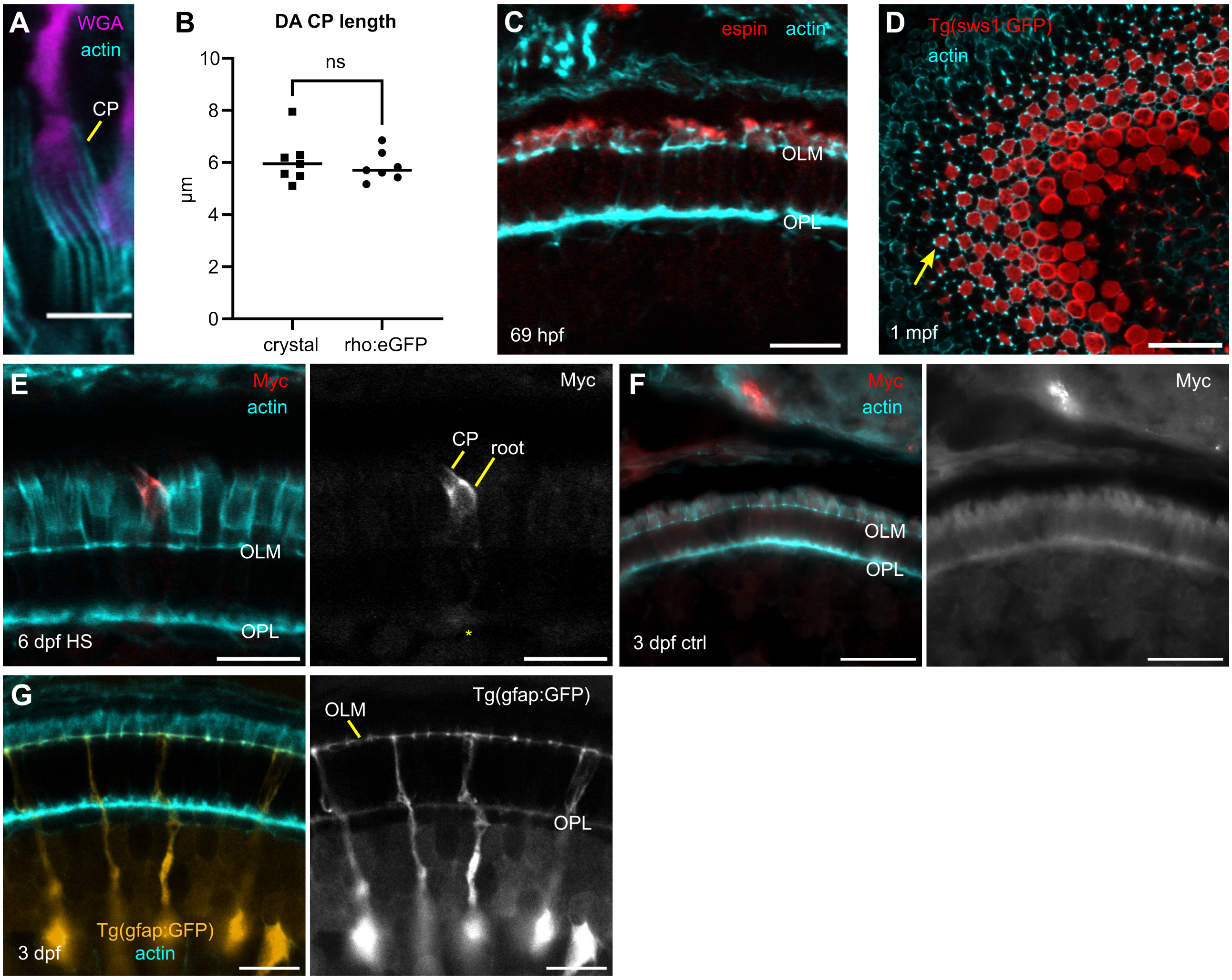

Fig. S2. (A) Confocal image of a 1 mpf crystal zebrafish photoreceptor layer stained with WGA and phalloidin, a single rod is shown. (B) Graph displaying the comparison between CP length in 1 mpf DA *crystal* fish and *Tg(rho:eGFP)*; n=7 in both groups median is shown; unpaired t-tests with Welch's correction; two-tailed p-value; ns—p>0.05. (C) Confocal image of 69 hpf outer retina sections stained with phalloidin (cyan) and anti-espin (red). (D) *Tg(sws1:GFP)* (GFP in red) 1 mpf zebrafish retina stained with phalloidin (cyan), sagittal section. Arrow is pointing at UVS OS surrounded by thick actin bundles. (E) Confocal images of 6 dpf Tol2/hsp70l:zact-MTpA-injected zebrafish 24 h after heat shock stained with phalloidin and anti-myc antibody. Actin-myc localization in the calyceal process and its root is highlighted. Asterisk indicates diffuse staining at the OPL. (F) Control 3 dpf *Tg(hsp:act-myc)* photoreceptor layer. (G) Confocal image of *Tg(gfap:GFP)* 3 dpf zebrafish retina (GFP shown in orange) stained with phalloidin (cyan); no glial processes above the OLM are observed. Number of fish analyzed n=7 (A), n=4 (C), n=6 (D), n=8 (E), n=5 (F), n=4 (G). Scale bars: 5 µm (A), 10 µm (C,E,G), 20 µm (D,F).

Journal of Cell Science • Supplementary information

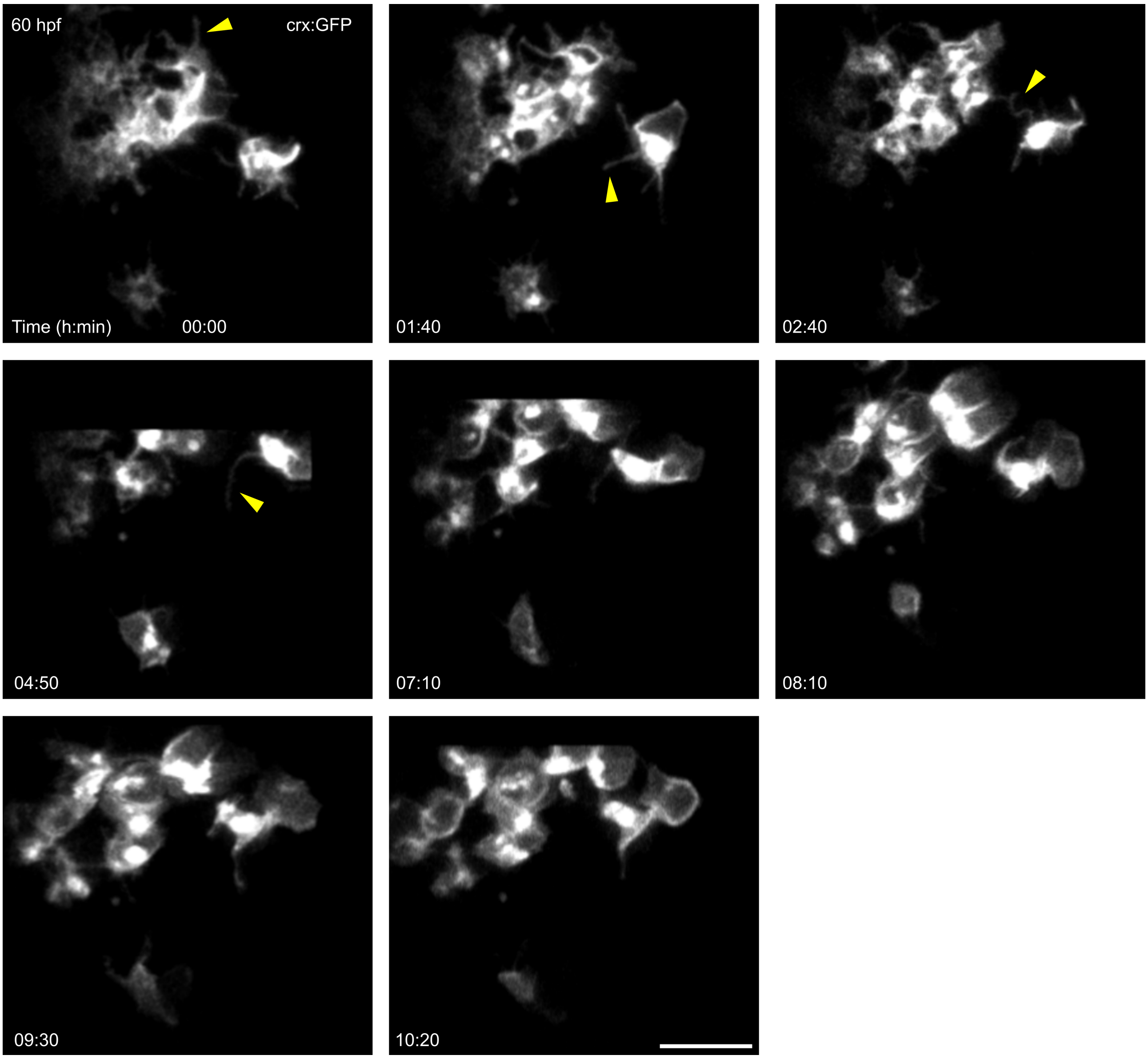

Fig. S3. Selected time points from Movie S3. Confocal time-lapse experiment (maximum intensity projection) of a *crx:GFP* transgenic embryo from 60 hpf to 72 hpf, apical view. Arrowheads highlight dynamic tangential processes sprouting (and retracting) from the apical surface of the photoreceptor progenitors. See Fig. 4A' for orientation. Scale bar: 10 µm.

Journal of Cell Science • Supplementary information

Table S1. List of labeling reagents for fluorescence microscopy.

Molecule type	Host	Fluorophore	Dilution	Supplier	Catalogue #
anti-espin antibody	rabbit	-	1:25	Bartles lab	-
anti-GFP antibody	rabbit	-	1:500	Invitrogen	A11122
anti-blue opsin antibody	rabbit	-	1:200	Kerafast	Q9W6A8
anti-UV opsin antibody	rabbit	-	1:200	Kerafast	EJH013
anti-myc 9E10 antibody	mouse	-	1:500	ATCC	CRL-1729
zpr1 antibody	mouse	-	1:100	ZIRC	zpr-1
zpr2 antibody	mouse	-	1:100	ZIRC	zpr-2
anti-mouse antibody	goat	Alexa Fluor 680	1:250	Invitrogen	A21057
anti-rabbit antibody	goat	Alexa Fluor 680	1:250	Invitrogen	A27042
anti-mouse antibody	donkey	Alexa Fluor 555	1:250	Invitrogen	A31570
anti-mouse antibody	donkey	Alexa Fluor 647	1:250	Invitrogen	A31571
anti-rabbit antibody	donkey	Alexa Fluor 546	1:250	Invitrogen	A10040
anti-rabbit antibody	goat	Alexa Fluor 488	1:250	Invitrogen	A11034
phalloidin	-	CF633	1:200	Biotium	00046
phalloidin	-	iFluor 488	1:1000	Cayman Chemical	20549
phalloidin	-	rhodamine	1:1000	Abcam	ab235138
phalloidin	-	TRITC	1:2500	Sigma-Aldrich	P1951
PNA lectin	-	Alexa Fluor 568	1:200	Invitrogen	L32458
WGA lectin	-	FITC	1:200	Sigma	L4895

Journal of Cell Science • Supplementary information

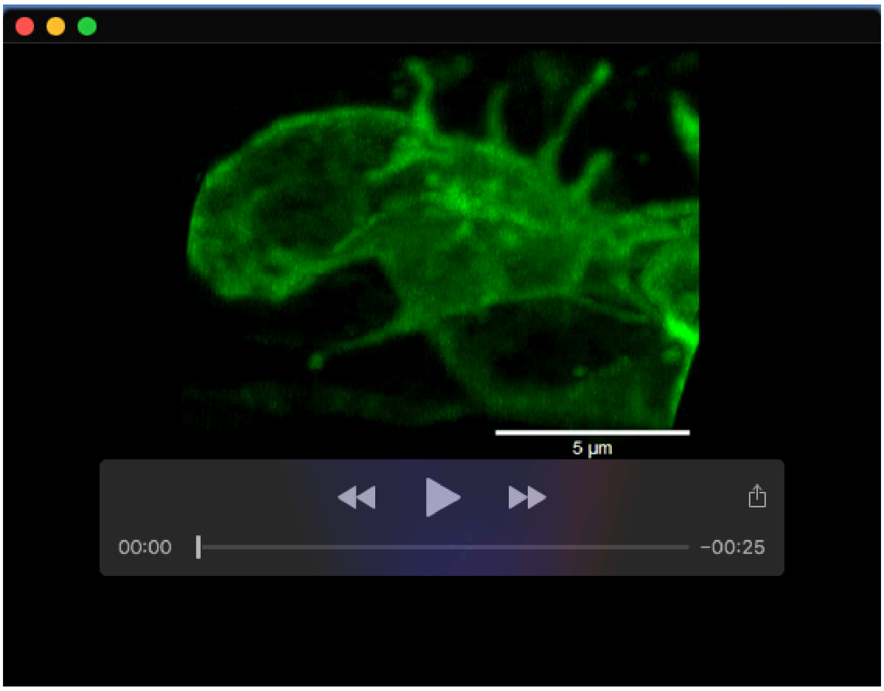

Movie 1. 3D reconstruction from a confocal stack at high magnification of the apical retina from a 72 hpf *crx:EGFP-CAAX* (*crx:GFP*, green) transgenic embryo, labeled with phalloidin (F-actin, cyan). Scale bar: 5 µm. See Fig. 4A for further reference.

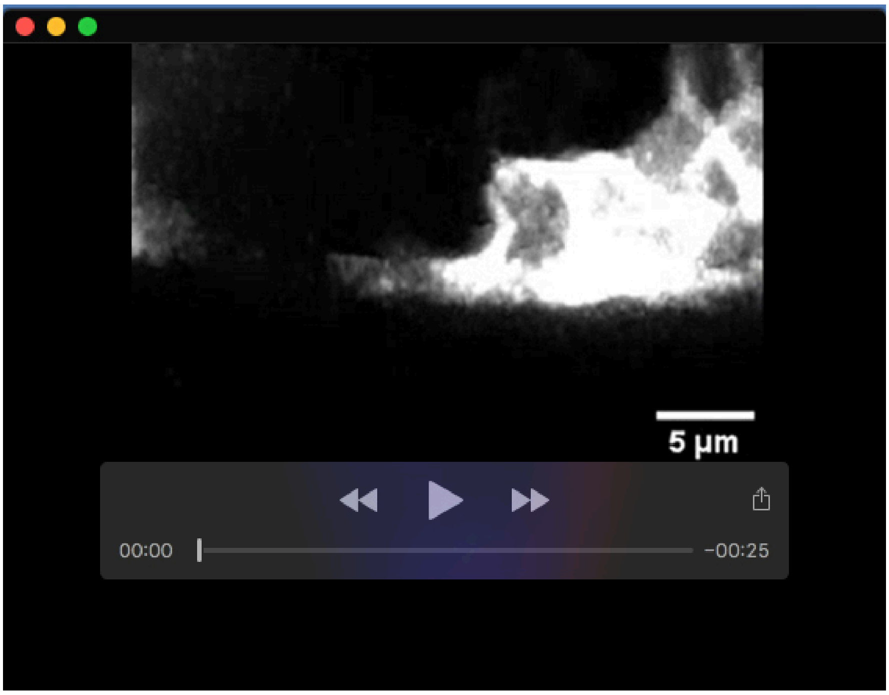

Movie 2. Confocal time-lapse experiment (maximum intensity projection) of a *crx:GFP* transgenic embryo from 60 hpf to 72 hpf, with a temporal resolution of 10 min. During this time, the imaged cells at the apical-most region of the retina display the extension and retraction of tangential processes. Scale bar: 5 µm. See Fig. 4A for further reference.

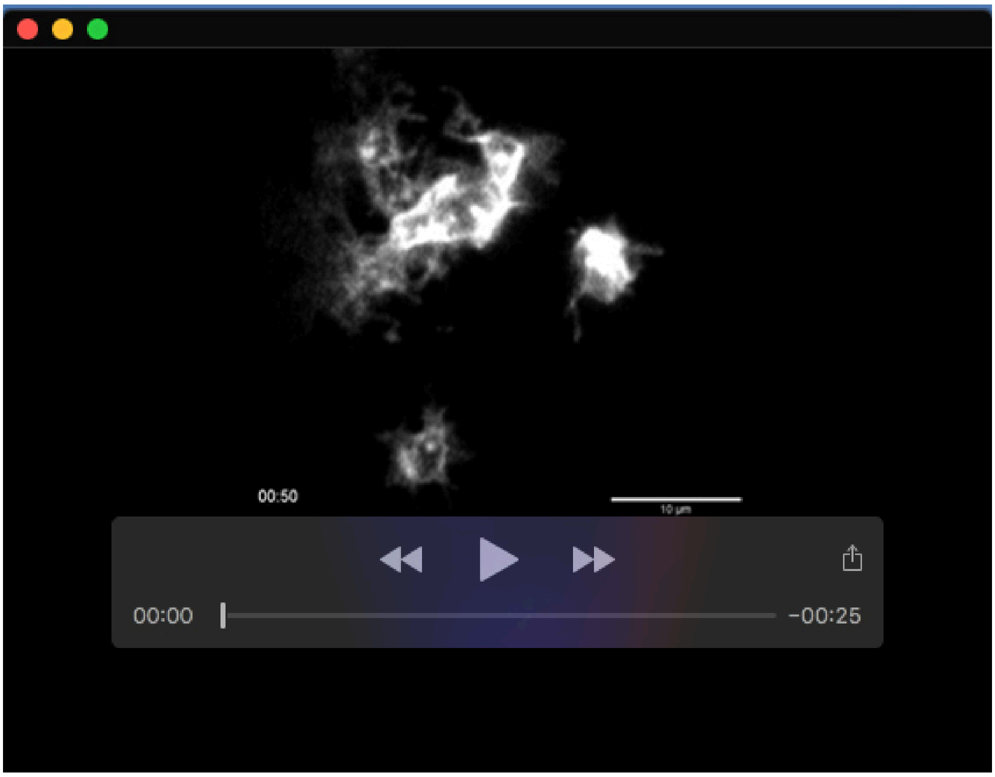

Movie 3. Confocal time-lapse experiment (maximum intensity projection) of a *crx:GFP* transgenic embryo from 60 hpf to 72 hpf, apical view. During this time, the imaged cells display the extension and retraction of tangential processes. Temporal resolution: 10 min. Scale bar: 10 μm. See Fig. S3 for selected time points.

Journal of Cell Science • Supplementary information
