# Supplementary material for: Photoreceptor calyceal processes accompany the developing outer segment, adopting a stable length despite a dynamic core
Source: J Cell Sci. 2024 Apr 12;137(7):jcs261721. doi: 10.1242/jcs.261721 (PMC11058337; doi:10.1242/jcs.261721)
Supplement: Supplementary file 1 [file joces-137-261721_review_history.pdf]

Photoreceptor calyceal processes accompany the developing outer segment, adopting a stable length despite a dynamic core

Maria Sharkova, Gonzalo Aparicio, Constantin Mouzaaber, Flavio R Zolessi and Jennifer C Hocking

DOI: 10.1242/jcs.261721

Editor: Caroline Hill

Review timeline
Original submission: 16 October 2023
Editorial decision: 15 December 2023
First revision received: 26 February 2024
Accepted: 4 March 2024

Original submission

First decision letter

MS ID#: JOCES/2023/261721

MS TITLE: Development and characteristics of photoreceptor actin-based apical processes in zebrafish (*Danio rerio*)

AUTHORS: Maria Sharkova, Gonzalo Aparicio, Constantin Mouzaaber, Flavio R Zolessi, and Jennifer C Hocking

ARTICLE TYPE: Research Article

We have now reached a decision on the above manuscript.

To see the reviewers' reports and a copy of this decision letter, please go to: https://submit-jcs.biologists.org and click on the 'Manuscripts with Decisions' queue in the Author Area. (Corresponding author only has access to reviews.)

As you will see, the reviewers gave favourable reports but raised some critical points that will require amendments to your manuscript. I hope that you will be able to carry these out because I would like to be able to accept your paper, depending on further comments from reviewers.

Please ensure that you clearly highlight all changes made in the revised manuscript. Please avoid using 'Tracked changes' in Word files as these are lost in PDF conversion.

I should be grateful if you would also provide a point-by-point response detailing how you have dealt with the points raised by the reviewers in the 'Response to Reviewers' box. Please attend to all of the reviewers' comments. If you do not agree with any of their criticisms or suggestions please explain clearly why this is so.

Reviewer 1

Advance summary and potential significance to field

© 2024. Published by The Company of Biologists under the terms of the Creative Commons Attribution License (https://creativecommons.org/licenses/by/4.0/).

The role of calyceal processes (CPs) in photoreceptor health and development is not well understood. Sharkova M et al, provide a comprehensive characterisation of the CPs in zebrafish by fluorescence and electron microscopy using a range of transgenic lines. This work includes difference in CPs morphology between photoreceptor types and the formation and maturation of CPs. Due to the CPs being associated with normal photoreceptor outer segment morphology and Usher syndrome this is important work.

Comments for the author

More information should be included about why the blue cones were excluded from the CP length and diameter analysis.

It would be good to include example electron microscopy images of DC and UVS photoreceptors and the associated CPs either in Figure 1 or in a supplementary figure.

In Figure 3 panels A and B, it is hard to see the processes and it would help to have a zoomed in panels similar to the example given in C?

In Figures 4&5 could some of the actin rich structures be Müller glial processes? Have you looked at the Tg(gfap:GFP) model at earlier timepoints?

Minor comment
Figure 5 - Legend mentions panels (B-F) but there is no F.
Figure 6 B - Need to include what the arrowhead represents.
Figure 7 - It looks odd having the cilium extend over the position of the RPE nucleus.

Reviewer 2

Advance summary and potential significance to field

In this manuscript, Sharkova et al characterize a number of properties of the calyceal processes and other “actin-based” processes of zebrafish retinal photoreceptors, and make several novel observations.

Calyceal processes are microvilli-like protrusions of the photoreceptor inner segment membrane that surround rod and cone outer segments (OS) like a basket.
They contain actin filaments and are readily labeled by phalloidin. They are analogous to the stereocilia of auditory hair cells, and Usher’s syndrome, which causes both deafness and blindness, involves defects in genes involve in creating or maintaining these structures. However, mice with mutations in USH genes do not develop visual defects; mice do not have calyceal processes. The role of calyceal processes in photoreceptor cell biology is not well understood.
Therefore, investigation of their properties in non-mouse systems may provide insight into both photoreceptor cell biology and Usher syndrome disease processes.

In Figure 1, the authors characterize the length and diameter of calyceal processes (CPs) in the different photoreceptor types of the zebrafish retina, as well as their relative lengths compared to OS length, and their diameters demonstrating that each photoreceptor type has unique calyceal process dimensions.

In Figure 2, the authors demonstrate that the various photoreceptor cell types of the zebrafish retina undergo retinomotor movements, but the length of CPs is unaffected by these movements (in contrast to previously published descriptions of the CPs of sunfish).

In Figure 3, the authors examine the development of CPs, showing that their development is preceded by the appearance of small villi-like structures on the apical plasma membrane, followed by elaboration of the OS cilia. CPs are not apparent until OS appear. An apical “actin dome” structure appears prior to the elaboration of OS and CP

© 2024. Published by The Company of Biologists under the terms of the Creative Commons Attribution License (https://creativecommons.org/licenses/by/4.0/).

In Figure 4, the authors use mosaic expression of membrane-attached GFP in photoreceptors to demonstrate that developing photoreceptors elaborate dynamic tangential processes from their apical membranes that briefly overlap with development of CPs; however, these appear to be distinct from CPs.

In Figure 5, the authors use heat-shock induced expression of myc-tagged actin to examine actin turnover in CPs. They conclude that in both developing and mature photoreceptors the actin within CPs rapidly turns over. This is unlike actin in stereocilia, in which only the tips of the actin bundles turn over rapidly. To me, this result was quite surprising.

In Figure 6, the authors show that muller glia extend processes that surround photoreceptor cell bodies (previously documented) and that in the UV sensitive cones, these processes extend as high as the OS where they contact RPE processes
(novel finding).

Figure 7 summarizes findings in a cartoon depicting CP development incorporating the authors' findings (but not the findings of figure 6).
Overall, the data is of high quality, the paper is well written, and the findings are novel. The paper adds significantly to our knowledge of the structure and development of CPs in a well-researched laboratory animal. I would imagine that this paper would likely be cited by many future studies of CPs in fish, as well as other species, and possibly this author has future studies planned in which the properties of CPs will be probed using this paper's characterizations as a reference point.

Comments for the author

One issue is that in the discussion there is no distinction of a specific finding described as critical or most important…however this is more of a style element that might vary from author to author.

I think the title could be improved. It does not mention "calyceal processes", which might limit readership. "Actin-based processes" is used because not all the structures studied are CPs. However, "CPs and other actin-based processes" might be preferable. Furthermore, although the Muller glia finding is mentioned in the abstract, it is not incorporated into the title (and that result involves actin-based processes of Muller cells, not photoreceptors). Perhaps the authors could also attempt to incorporate the MG finding into the title.

Overall the paper is well written and needs minimal editing. A few points are noted below:

Top of page 6 "Peripherally, photoreceptors were…" would be better worded as "Peripheral photoreceptors were…"

Section 2.4: cells are described as "crx-positive" but this phrase would normally be used to refer to antibody labeling for crx…rather, these cells express a transgene from the CRX promoter, so more correctly, they are GFP-positive. Another alternative would be "cells with CRX promoter-driven GFP expression" or similar.

Top of page 11: "cells that are noticeably taller…" should be "cells that are noticeably longer…"

Table 1: "list of staining molecules…" should be "list of labeling reagents for fluorescence microscopy" or similar.

Methods: a "Zeiss Elyra" microscope is mentioned as being used to resolve "fine details" in the tagged actin experiment, but it is not clear what type of microscope this is. Is it a SIM, or similar superresolution microscope? If all other images are standard confocal, it would be best to say "used to resolve fine detail for experiments reported in figure X.Y-Z"

Figure 1 legend: "F, G Example of TEM…." Should be "F, F', example of TEM…"

© 2024. Published by The Company of Biologists under the terms of the Creative Commons Attribution License (https://creativecommons.org/licenses/by/4.0/).

Reviewer 3

Advance summary and potential significance to field

Although CP's were described over 100 years ago, and are known to be associated with photoreceptor outer segments in several vertebrate species (including humans), their function in supporting vision is still unknown. Even less is known about their development, or whether/how CP length is regulated concomitantly with circadian and other homeostatic fluctuations in photoreceptor outer segment length. The biomedical importance of these questions is underscored by diseases such as Usher syndrome, in which the structure of CP's is disrupted. Therefore, a better understanding of CP structure, development, and regulation would provide an important foundation for future perturbation studies, and is a necessary first step in elucidating their function. I believe this study will be of strong interest to those in the field studying photoreceptor structure, outer segment morphogenesis and maintenance, and to those interested in the pathogenesis of Usher syndrome.

Comments for the author

The study by Sharkova et al presents a detailed description of the development and morphology of photoreceptor calyceal processes (CP's) in zebrafish. Although CP's were described over 100 years ago, and are known to be associated with photoreceptor outer segments in several vertebrate species (including humans), their function in supporting vision is still unknown. Even less is known about their development, or whether/how CP length is regulated concomitantly with circadian and other homeostatic fluctuations in photoreceptor outer segment length. The biomedical importance of these questions is underscored by diseases such as Usher syndrome, in which the structure of CP's is disrupted. Therefore, a better understanding of CP structure, development, and regulation would provide an important foundation for future perturbation studies, and is a necessary first step in elucidating their function. Here, Sharkova et al use a variety of imaging techniques (confocal, TEM, in vivo time-lapse imaging) combined with transgenic labeling of cell types/structures and immunohistochemistry to document the emergence of the CP in zebrafish photoreceptors, as well as differences in CP dimensions between cone subtypes. The manuscript is well written, the experiments are carefully and rigorously performed, the images are of excellent quality, and the analysis and interpretation of the results is thoughtful. One of the most interesting findings (in my opinion) is that CP length appears to stay constant even when photoreceptor OS length changes (for example, during retinomotor movements associated with light and dark adaptation). This result suggests that CPs are not just passive appendages to the OS, but have their own distinct mechanisms for regulating their structure. I believe this study will be of strong interest to those in the field studying outer segment morphogenesis and maintenance, and to those interested in the pathogenesis of Usher syndrome. While the experiments were not designed to provide a mechanistic understanding of the phenomenon under study, I feel it would be premature and unreasonable to expect data of that sort at this early stage in our understanding of CP biology. Therefore, I feel comfortable recommendation publication subsequent to minor revisions. To that end, I have just a few suggestions (detailed below) for clarification of the text and figures.

1) Results and Figure 1B/C: are the length differences statistically significant? This isn't mentioned or indicated on the figure
2) Figure 3A-B: the "periphery" label doesn't seem to apply to 3B, since the Figure legend describes this image as one taken from "moving away from the periphery"
3) Results/Figure 5: Could the dynamic incorporation of actin at the CP base while maintaining constant length could suggest turnover at the CP tip (similar to OS disk shedding)? Other interpretations of this observation?
4) Results/Figure 6: The observation of interaction between the apical processes of the Muller glia and the tips of the RPE is interesting and novel, but doesn't relate to the central topic of the study, the CP. Is there any evidence of interaction between the UVS CP and the MG apical process?

© 2024. Published by The Company of Biologists under the terms of the Creative Commons Attribution License (https://creativecommons.org/licenses/by/4.0/).

First revision

Author response to reviewers' comments

Response to Reviewers

We thank the three reviewers for taking the time to read our manuscript and for providing positive comments and helpful suggestions. Please find our detailed responses below.
Reviewer 1 Advance Summary and Potential Significance to Field:
The role of calyceal processes (CPs) in photoreceptor health and development is not well understood. Sharkova M et al, provide a comprehensive characterisation of the CPs in zebrafish by fluorescence and electron microscopy using a range of transgenic lines. This work includes difference in CPs morphology between photoreceptor types and the formation and maturation of CPs. Due to the CPs being associated with normal photoreceptor outer segment morphology and Usher syndrome this is important work.

Reviewer 1 Comments for the Author:
More information should be included about why the blue cones were excluded from the CP length and diameter analysis.

We obtained a blue cone antibody and have now completed an analysis of blue cone CPs. Please see the additional data in Figures 1 and 2.

It would be good to include example electron microscopy images of DC and UVS photoreceptors and the associated CPs either in Figure 1 or in a supplementary figure.

The requested TEM images have been added in Supplementary Figure 1.

In Figure 3 panels A and B, it is hard to see the processes and it would help to have a zoomed in panels similar to the example given in C?

The figure was reorganized and each TEM image in Figure 3 now shows a more focused view. We moved panel C’ from the original figure to Supplementary Figure 1. We hope the reviewer will now find the data easier to visualize.

In Figures 4&5 could some of the actin rich structures be Müller glial processes? Have you looked at the Tg(gfap:GFP) model at earlier timepoints?

This is a good point given the presence of long glial processes protruding into the photoreceptor layer of older fish. We were confident that we were analyzing processes extending directly from photoreceptor cells as we could see direct connections to the actin dome. Nevertheless, we performed phalloidin staining on 72 hpf Tg(gfap:GFP) fish and confirmed that there are no glial processes present alongside the developing photoreceptor inner segments. This data has now been added to the manuscript as Supplemental Figure 2G.

Minor comment
Figure 5 - Legend mentions panels (B-F) but there is no F.

Correction was made.

Figure 6 B - Need to include what the arrowhead represents.

Correction was made.

Figure 7 - It looks odd having the cilium extend over the position of the RPE nucleus.

We agree with the reviewer comment. The image is not to scale given that the RPE interacts with many photoreceptors. We have removed the nucleus to indicate that the RPE is a portion of a cell. The figure legend has been updated to explain this: “Please note that the diagram does not

© 2024. Published by The Company of Biologists under the terms of the Creative Commons Attribution License (https://creativecommons.org/licenses/by/4.0/).

accurately depict the relative sizes of the photoreceptors and RPE in order to highlight the apical region of the former."

*****

Reviewer 2 Advance Summary and Potential Significance to Field:
In this manuscript, Sharkova et al characterize a number of properties of the calyceal processes and other "actin-based" processes of zebrafish retinal photoreceptors, and make several novel observations.

Calyceal processes are microvilli-like protrusions of the photoreceptor inner segment membrane that surround rod and cone outer segments (OS) like a basket.
They contain actin filaments and are readily labeled by phalloidin. They are analogous to the stereocilia of auditory hair cells, and Usher's syndrome, which causes both deafness and blindness, involves defects in genes involve in creating or maintaining these structures. However, mice with mutations in USH genes do not develop visual defects; mice do not have calyceal processes. The role of calyceal processes in photoreceptor cell biology is not well understood.
Therefore, investigation of their properties in non-mouse systems may provide insight into both photoreceptor cell biology and Usher syndrome disease processes.

In Figure 1, the authors characterize the length and diameter of calyceal processes (CPs) in the different photoreceptor types of the zebrafish retina, as well as their relative lengths compared to OS length, and their diameters, demonstrating that each photoreceptor type has unique calyceal process dimensions.

In Figure 2, the authors demonstrate that the various photoreceptor cell types of the zebrafish retina undergo retinomotor movements, but the length of CPs is unaffected by these movements (in contrast to previously published descriptions of the CPs of sunfish).

In Figure 3, the authors examine the development of CPs, showing that their development is preceded by the appearance of small villi-like structures on the apical plasma membrane, followed by elaboration of the OS cilia. CPs are not apparent until OS appear. An apical "actin dome" structure appears prior to the elaboration of OS and CP

In Figure 4, the authors use mosaic expression of membrane-attached GFP in photoreceptors to demonstrate that developing photoreceptors elaborate dynamic tangential processes from their apical membranes that briefly overlap with development of CPs; however, these appear to be distinct from CPs.

In Figure 5, the authors use heat-shock induced expression of myc-tagged actin to examine actin turnover in CPs. They conclude that in both developing and mature photoreceptors the actin within CPs rapidly turns over. This is unlike actin in stereocilia, in which only the tips of the actin bundles turn over rapidly. To me, this result was quite surprising.

In Figure 6, the authors show that muller glia extend processes that surround photoreceptor cell bodies (previously documented) and that in the UV sensitive cones, these processes extend as high as the OS where they contact RPE processes (novel finding).

Figure 7 summarizes findings in a cartoon depicting CP development, incorporating the authors' findings (but not the findings of figure 6).
Overall, the data is of high quality, the paper is well written, and the findings are novel. The paper adds significantly to our knowledge of the structure and development of CPs in a well-researched laboratory animal. I would imagine that this paper would likely be cited by many future studies of CPs in fish, as well as other species, and possibly this author has future studies planned in which the properties of CPs will be probed using this paper's characterizations as a reference point.

We thank the reviewer for the kind comments

Reviewer 2 Comments for the Author:
One issue is that in the discussion there is no distinction of a specific finding described as critical or most important…however this is more of a style element that might vary from author to author.

© 2024. Published by The Company of Biologists under the terms of the Creative Commons Attribution License (https://creativecommons.org/licenses/by/4.0/).

I think the title could be improved. It does not mention “calyceal processes”, which might limit readership. “Actin-based processes” is used because not all the structures studied are CPs. However, “CPs and other actin-based processes” might be preferable. Furthermore, although the Muller glia finding is mentioned in the abstract, it is not incorporated into the title (and that result involves actin-based processes of Muller cells, not photoreceptors). Perhaps the authors could also attempt to incorporate the MG finding into the title.

We agree with the reviewer comments that we did not strongly highlight one finding in the discussion or the title. That came from the nature of the paper, as it covered many different points that together provide a clear picture of the development, characteristics, and surrounding environment of the calyceal processes. However, upon the reviewer’s suggestion, we have made some changes to the discussion to strengthen the emphasis on our findings and have decided on a new title: Photoreceptor calyceal processes accompany the developing outer segment, adopting a stable length despite a dynamic core. Note, we decided not to incorporate the MG finding in the title because it was not the main topic of the paper and we are instead pursing a second study investigating MG processes and MG-RPE, MG-POS interactions.

Overall the paper is well written and needs minimal editing. A few points are noted below:

Thank you

Top of page 6 “Peripherally, photoreceptors were…” would be better worded as “Peripheral photoreceptors were…”

Correction was made as suggested.

Section 2.4: cells are described as “crx-positive” but this phrase would normally be used to refer to antibody labeling for crx…rather, these cells express a transgene from the CRX promoter, so more correctly, they are GFP-positive. Another alternative would be “cells with CRX promoter-driven GFP expression” or similar.

Changes were made so now we describe the cells as “GFP-positive cells” or “cells with CRX promoter-driven GFP expression”

Top of page 11: “cells that are noticeably taller…” should be “cells that are noticeably longer…”

Correction was made as suggested.

Table 1: “list of staining molecules…” should be “list of labeling reagents for fluorescence microscopy” or similar.

Correction was made as suggested.

Methods: a “Zeiss Elyra” microscope is mentioned as being used to resolve “fine details” in the tagged actin experiment, but it is not clear what type of microscope this is. Is it a SIM, or similar superresolution microscope? If all other images are standard confocal, it would be best to say “used to resolve fine detail for experiments reported in figure X.Y-Z”

Description of the microscope was changed to “Zeiss Elyra lattice SIM”

Figure 1 legend: “F, G Example of TEM….” Should be “F, F’, example of TEM…

Correction was made as suggested.

Reviewer 3
The study by Sharkova et al presents a detailed description of the development and morphology of photoreceptor calyceal processes (CP’s) in zebrafish. Although CP’s were described over 100 years ago, and are known to be associated with photoreceptor outer segments in several vertebrate species (including humans), their function in supporting vision is still unknown. Even less is known

© 2024. Published by The Company of Biologists under the terms of the Creative Commons Attribution License (https://creativecommons.org/licenses/by/4.0/).

about their development, or whether/how CP length is regulated concomitantly with circadian and other homeostatic fluctuations in photoreceptor outer segment length. The biomedical importance of these questions is underscored by diseases such as Usher syndrome, in which the structure of CP's is disrupted. Therefore, a better understanding of CP structure, development, and regulation would provide an important foundation for future perturbation studies, and is a necessary first step in elucidating their function. Here, Sharkova et al use a variety of imaging techniques (confocal, TEM, in vivo time-lapse imaging) combined with transgenic labeling of cell types/structures and immunohistochemistry to document the emergence of the CP in zebrafish photoreceptors, as well as differences in CP dimensions between cone subtypes. The manuscript is well written, the experiments are carefully and rigorously performed, the images are of excellent quality, and the analysis and interpretation of the results is thoughtful. One of the most interesting findings (in my opinion) is that CP length appears to stay constant even when photoreceptor OS length changes (for example, during retinomotor movements associated with light and dark adaptation). This result suggests that CPs are not just passive appendages to the OS, but have their own distinct mechanisms for regulating their structure. I believe this study will be of strong interest to those in the field studying outer segment morphogenesis and maintenance, and to those interested in the pathogenesis of Usher syndrome. While the experiments were not designed to provide a mechanistic understanding of the phenomenon under study, I feel it would be premature and unreasonable to expect data of that sort at this early stage in our understanding of CP biology. Therefore, I feel comfortable recommendation publication subsequent to minor revisions. To that end, I have just a few suggestions (detailed below) for clarification of the text and figures.

We thank the reviewer for their kind comments and interest in our research.

1) Results and Figure 1B/C: are the length differences statistically significant? This isn't mentioned or indicated on the figure.

We have now included statistics comparing the length differences between photoreceptor subtypes. Note that blue cone measurements have been added as per request from Reviewer 1.

2) Figure 3A-B: the "periphery" label doesn't seem to apply to 3B, since the Figure legend describes this image as one taken from "moving away from the periphery"

Labels were removed and now we have added a schematic to better convey the image location.

3) Results/Figure 5: Could the dynamic incorporation of actin at the CP base while maintaining constant length could suggest turnover at the CP tip (similar to OS disk shedding)? Other interpretations of this observation?

We agree that CPs somehow must constantly adapt to the growing OS. However, our actin incorporation experiment only reveals the dynamic nature of the CP actin core, but does not elucidate the exact pattern of turnover. Further, we hypothesize that it occurs in a treadmilling manner, as per intestinal microvilli, which would involve addition of actin monomers at the tip and removal from the roots. Continual shedding of the tip would therefore not fit with such a model of actin dynamics, although we would not rule it out. We have added the following statement to the discussion:
"Treadmilling involves the addition of actin monomers to the F-actin plus ends at the microvillar tips and removal from the cytosolic minus ends. Using our heat shock system, we showed rapid turnover in CPs but does not elucidate the exact pattern of actin monomer addition and removal. However, the actin bundle in CPs is reportedly oriented as in other microvilli, with the plus ends at the distal tip, suggesting a similar mechanism of actin renewal."

4) Results/Figure 6: The observation of interaction between the apical processes of the Muller glia and the tips of the RPE is interesting and novel, but doesn't relate to the central topic of the study, the CP. Is there any evidence of interaction between the UVS CP and the MG apical process?

We understand the reviewer's opinion that the MG finding is outside the main topic of the paper. However, it provides important context, as we found a far more intricate support network for the UVS cone outer segments than the basal ring of CPs we expected. We are currently following up on our MG findings in a second study, but we do feel its important to report our observations

© 2024. Published by The Company of Biologists under the terms of the Creative Commons Attribution License (https://creativecommons.org/licenses/by/4.0/).

immediately as we can clearly see in the literature that the glial presence in the photoreceptor layer is underappreciated. Note, we recently found a study from 1964 that showed CP-MG contact in frogs (Sven Erik G. Nilsson, An electron microscopic classification of the retinal receptors of the leopard frog (Rana pipiens), Journal of Ultrastructure Research, Volume 10, Issues 5–6, 1964, 390-416, doi.org/10.1016/S0022-5320(64)80018-6.citation was added to the text), highlighting that our observation has relevance beyond zebrafish.
Beyond close apposition, there is not yet evidence of an interaction between CPs and the MG apical processes. This would be a fascinating topic for further investigation.

Second decision letter

MS ID#: JOCES/2023/261721

MS TITLE: Photoreceptor calyceal processes accompany the developing outer segment, adopting a stable length despite a dynamic core

AUTHORS: Maria Sharkova, Gonzalo Aparicio, Constantin Mouzaaber, Flavio R Zolessi, and Jennifer C Hocking

ARTICLE TYPE: Research Article

I am happy to tell you that your manuscript has been accepted for publication in Journal of Cell Science, pending standard ethics checks.

© 2024. Published by The Company of Biologists under the terms of the Creative Commons Attribution License (https://creativecommons.org/licenses/by/4.0/).
